# Chemical Composition and Antimicrobial Activity of Two Sri Lankan Lichens, *Parmotrema rampoddense*, and *Parmotrema tinctorum* against Methicillin-Sensitive and Methicillin-Resistant *Staphylococcus aureus*

**DOI:** 10.1155/2021/9985325

**Published:** 2021-06-01

**Authors:** Polwatta Samaraweera Arachchige Ishara Shiromi, Ruwani Punyakanthi Hewawasam, Rankoth Gedara Udeni Jayalal, Hasanga Rathnayake, Weerasinghe Mudiyanselage Dilip Gaya Bandara Wijayaratne, Dakshika Wanniarachchi

**Affiliations:** ^1^Department of Natural Resources, Faculty of Applied Sciences, Sabaragamuwa University of Sri Lanka, Belihuloya, Sri Lanka; ^2^Department of Biochemistry, Faculty of Medicine, University of Ruhuna, Galle, Sri Lanka; ^3^Department of Microbiology, Faculty of Medicine, University of Ruhuna, Galle, Sri Lanka; ^4^Instrument Center, Faculty of Applied Sciences, University of Sri Jayewardenepura, Nugegoda, Sri Lanka

## Abstract

**Introduction:**

Medicinal utility of lichens is ascribed to the presence of various secondary metabolites of low molecular weight and they have been used in traditional medicine including Ayurveda in the treatment of wounds and skin disorders. Despite the urgent need to effectively address the antibiotic resistance worldwide, the discovery of new antibacterial drugs has declined in the recent past. This emphasizes the increasing importance of investigating and developing new classes of antibiotics that can withstand antibiotic resistance. *Aims of the study*. The present study was conducted to investigate the chemical composition and the antibacterial activity of hexane, ethanol, and aqueous extracts of *Parmotrema rampoddense* and *Parmotrema tinctorum*, two lichens collected from Belihuloya, Sri Lanka, against Gram-negative and Gram-positive bacteria including twenty clinical isolates of methicillin-resistant *Staphylococcus aureus* (MRSA). *Materials and methods*. Phytochemical analysis, thin layer chromatography (TLC), and Gas Chromatography Mass Spectrometry (GC-MS) were performed to determine the chemical composition of the two lichens. Hexane, ethanol, and aqueous extracts of both lichens were tested against clinical isolate of Gram-negative and Gram-positive bacteria including twenty clinical isolates of MRSA. Bacterial susceptibility was tested using a disc diffusion assay. Minimum inhibitory concentration (MIC) was determined by a broth microdilution method. Vancomycin was used as the positive control.

**Results:**

Alectorialic acid, atranorin, atraric acid, orcinol, and O-orsellinaldehyde were among the secondary metabolites identified by the TLC and GC-MS analysis. None of the lichen extracts were active against Gram-negative bacteria but both lichens showed a concentration-dependent activity against methicillin-sensitive *Staphylococcus aureus* (MSSA) and MRSA. Ethanol extract of *P. rampoddense* showed the highest activity against MSSA with the MIC, 0.0192 mg/ml, but all MRSA isolates investigated showed MIC between 0.096 and 2.4 mg/ml for the same extract.

**Conclusion:**

Both lichens, *P. rampoddense* and *P. tinctorum*, represent potentially important sources of future antimicrobial drugs. Further investigation on the ethanol extract of *P. rampoddense* will enable us to determine the most active phytoconstituents responsible for the activity, their mechanism of action against bacterial pathogens, and also their cytotoxicity against normal cells.

## 1. Introduction

The recurrence of high-profile, multidrug-resistant (MDR) pathogens is a growing concern as global mortality rates from bacterial infections continue to increase worldwide [1]. *Staphylococcus aureus* is the primary causative agent of community acquired skin and soft tissue infections, and it is also an important cause of hospital-associated invasive infections including bacteremia, pneumonia, sepsis, and endocarditis. Despite the urgent need to effectively address antibiotic resistance, the discovery of new antibacterial drugs has declined in recent years. This is largely due to the resistance developed by pathogens long before many drugs come to the market. Furthermore, microbial resistance has severely impacted a drug's long-term potential to return a profit [2]. These issues emphasize the increasing importance of investigating and developing new classes of antibiotics that can withstand pathogen resistance or express a new mode of action, thereby increasing the potential lifetime of the antibacterial agents [3].

Sri Lanka is considered as a biodiversity hotspot in the world due to its diverse and luxuriant ecosystems [4]. Approximately 30% of Sri Lanka is covered by forest and the island is home to around 3000 angiosperm species and one quarter of it are endemic [5]. Also, Sri Lanka harbors a highly diverse lichen flora, approximately 1200 of which have already been identified. It is expected that 2000 or more lichen species could be found on the island nation due to the minimal knowledge of Sri Lankan lichen species [6]. Lichens are widely used in folk medicine in many countries, such as India, Sri Lanka, and the Ayurveda as well as Unani systems of medicine describe their use in the treatment of a multitude of diseases [7]. They are symbiotic systems consisting of a fungus and either an algae or a cyanobacterium. They represent one of the more promising potential sources of low molecular weight secondary compounds and more than 1000 different secondary compounds have already been reported from lichens and their cultured mycobionts [8].

Metabolites isolated from lichens exert a plethora of biological actions including antibiotic, analgesic, antimycotic, antiviral, anti-inflammatory, and cytotoxic activities [9, 10]. The activity of lichen derived compounds has also been proven against some MDR pathogenic bacteria including vancomycin-resistant enterococci and methicillin-resistant *Staphylococcus aureus* (MRSA) [11, 12]. Atranorin, salazinic acid, lecanoric acid, and usnic acid are well-known active constituents isolated from lichens and are reported to possess strong biological activities such as antibacterial, antifungal, antitumor, and antioxidant activities. Usnic acid, the most investigated metabolite with proven antibiotic activity, has been used in the development of pharmaceuticals and cosmetics. Recently, derivatives of usnic acid were synthesized which enhanced its antimicrobial activity [13]. According to literature, approximately 8% of the terrestrial ecosystem comprises lichens; thus, around 20,000 lichens are distributed throughout the world. However, their biological activities and characterization of active constituents remain unexplored to a significant extent [14].

**Figure 1:**
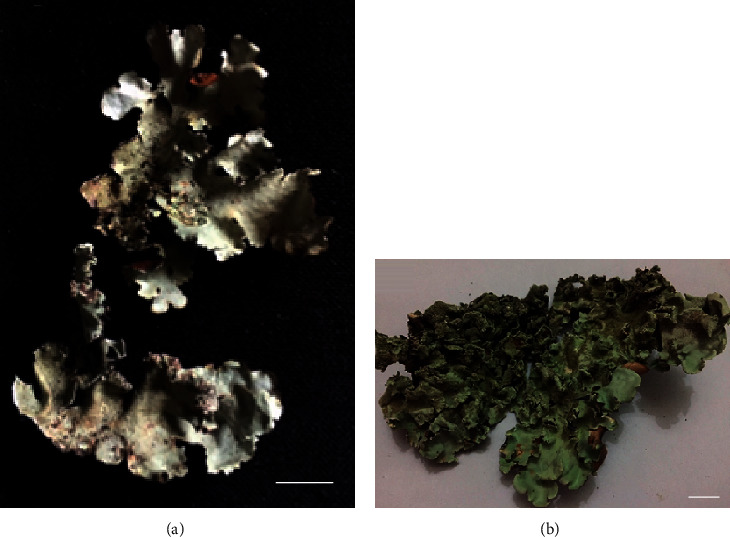
Lichens collected from Belihuloya, Sri Lanka. (a) *P. rampoddense* and (b) *P. tinctorum*, (scale bars: 1 cm).

This is the first detailed report of the chemical composition and the comparison of antimicrobial activity of aqueous, hexane, and ethanol extracts of *Parmotrema rampoddense* and *Parmotrema tinctorum* against CLSI (Clinical & Laboratory Standards Institute) standard strain of methicillin-sensitive *Staphylococcus aureus*, ATCC 25923 (MSSA), and clinical isolates of MRSA. *P rampoddense*, widely known as the “long-whiskered ruffle lichen,” is a species of the family Parmeliaceae (Figure 1(a)). It is widely distributed in tropical regions and grows on the bark of oak and palm trees. It has a largely expanded thallus and the thallus attaches to the substratum loosely. The upper surface of the thallus is mineral gray or whitish gray. The lower surface is black and wrinkled. The upper surface is rugose, smooth, and soredia are present. The lobes of the thallus are ciliated. This species has a corticolous habitat [15].

*P. tinctorum* (Figure 1(b)) has a thallus which is loosely attached to the substratum. The thallus is membranaceous to leathery. Irregular lobes are present in the thallus. Margins are either entire or scalloped. The upper surface has a gray color of a frosted look from a powdery coating (glaucous). The thallus is smooth, and marginal regions are folded longitudinally. The lower surface is black and wrinkled. This species can be easily distinguished from the other Parmeliaceae family species due to the loosely and largely arranged thallus, which is broad, ecilliated, and laminally isidiate lobes [15].

Both lichens have been used by the traditional ayurvedic medical practitioners in Sri Lanka in the treatment of wounds and various infectious diseases. Therefore, the present study was conducted to investigate the chemical composition of *P. rampoddense* and *P. tinctorum* collected from Belihuloya, Sri Lanka, by Thin layer chromatography (TLC) and Gas Chromatography-Mass Spectrophotometry (GC-MS) analysis and the antibacterial activity of hexane, ethanol, and aqueous extracts of each lichen against Gram-negative and Gram-positive bacteria including twenty clinical isolates of MRSA.

## 2. Materials and Methods

### 2.1. Collection of Lichen Species

Lichen samples of *P. rampoddense* (6°45′37″ °N 80°47′24″ °E) and *P. tinctorum* (6°45′38″ °N 80°47′23″ °E) were collected from the Nonpareil Estate, Belihuloya, Sri Lanka with permission of related authorities. Voucher specimens were deposited at the lichen herbarium of the Department of Natural Resources, Faculty of Applied Sciences, Sabaragamuwa University of Sri Lanka (Specimen No SUSLNR20 and SUSLNR21).

### 2.2. Identification of Lichens

The lichen samples were identified using stereo and light microscopes. Specifically, a dissecting microscope (LABOMED CZM6) was used to identify morphological characteristics of the thallus and the reproductive structures, while a compound microscope (Bio Blue 110–240v/50–60 Hz) was used to study the anatomy of thalli and fruiting bodies. Spot test reactions were carried out on thalli under the dissecting microscope.

### 2.3. Extraction of Lichen Metabolites

Each lichen thalli was washed thoroughly to remove debris in tap water and distilled water, oven dried at 40°C, and coarsely powdered. A cold extraction procedure was followed for the preparation of hexane and ethanol extracts. From each powdered lichen, 10 g was mixed with 100 ml of the solvent. The contents were placed in a mechanical shaker for 72 h at 25°C. Then, the extracts were filtered through Whattman No 1 filter paper, and the extract was concentrated at 40°C using a rotary evaporator (Hahnvapor, Hahnshin Scientific Co, HS-2005V-N). The concentrated extract was transferred to a pre-weighed sterile glass container and allowed to dry under a stream of air. The resulting crude extracts were dissolved in a minimum amount of 10% dimethyl sulfoxide (DMSO) separately, and the working stock solution was obtained by redissolving in 10% DMSO to yield a final concentration of 300 mg/ml for each extract. Each extract was stored at 4°C in sterile airtight containers in the dark for further studies.

The aqueous extract was prepared using 2.5 g of dried, coarsely powdered lichen refluxed in 30 ml of distilled water for 3 h. The resulting crude extract was dried at 40°C and was dissolved in a minimum amount of 10% DMSO, and the final concentration of the extract was adjusted to 300 mg/ml. The extract was stored at 4°C in sterile airtight containers in the dark for further studies.

### 2.4. Identification of Secondary Metabolites of Lichen Extracts

#### 2.4.1. Phytochemical Analysis of Lichen Extracts

A qualitative phytochemical analysis was carried out to identify the major classes of phytochemicals (tannins, alkaloids, phenolic compound, cyanogenic glycosides, cardiac glycosides, anthracene glycosides, reducing sugars, saponins, and flavonoids) present in various aqueous and alcoholic extracts of the two lichens by the methods described by Trease and Evans [16] and Sofowora [17]. Precautions were taken to remove the interference from chlorophyll before the commencement of the experiment.

#### 2.4.2. Identification of Lichen Metabolites by Thin-Layer Chromatography (TLC)

TLC fingerprint was obtained using the solvent system C (toluene: acetic acid = 170 : 30), which showed a fine separation with a maximum number of components. Two control samples including *Parmelia caperata* and known metabolites isolated from lichens were used as controls. Bands generated by constituents that could not be detected in the visible region were visualized under UV light at 254 nm and 366 nm. TLC plate was also sprayed with 10 % H_2_SO_4_ and subjected to heating at 110–120°C for 10 min before the observation for color changes and measurement of Rf value of the spots observed. The relative Rf (Retention factor) values for the chemicals extracted from both species were compared with the published literature to identify the specific compounds in each extract [18]. The experiment was performed in duplicate.

#### 2.4.3. GC-MS Analysis

GC–MS analyses were performed on an Agilent 7890 A/5975C-GC/MSD inert detector operating in EI mode with capillary-column chromatography HP-5 ms (30 m × 250 *μ*m × 0.25 *μ*m). The autosampler was used to inject 1 *μ*l of the sample with a 10 : 1 split ratio. The temperature of the injection port was 250°C. Temperature programming was conducted with the starting column temperature 70°C, which was held for 4 min, and then increased to 270°C at a rate of 10°C/min, held for 20 min. Helium was used as a carrier gas at a flow rate of 1.0 ml/min. The ionic source temperature was 230°C, the quadruple temperature was 150°C, and the scanning quality range was set at 50–550 amu. The percentage composition of the extracts was computed from the GC peak areas. Qualitative analysis on the identification of compounds was carried out using the NIST standard spectral library (https://www.nist.gov/). Nonisothermal retention indices were calculated using the definition of Van den Dool and Kratz [19].

(1) $$\begin{array}{c} \text{RI}=100n+\frac{100\left(t_{x}-t_{n}\right)}{\left(t_{n+1}-t_{n}\right)}, \end{array}$$

where *t*_*n*_ and *t*_*n*+1_ are retention times of the reference n-alkane hydrocarbons eluting immediately before and after the chemical compound “*X*.” The retention time of the compound “*X*” is given in *t*. Here, standard n-alkane series containing C_8_–C_20_ hydrocarbons (40 mg/L, Sigma-Aldrich)) in hexane was used for calculating the retention indices.

### 2.5. Determination of the Antimicrobial Activity of Lichen Extracts

#### 2.5.1. Microbial Strains

Both lichens were first screened against CLSI (Clinical and Laboratory Standards Institute) control strains of three Gram-negative bacteria, *Escherichila coli* (ATCC 25922), *Pseudomonas aeruginosa* (ATCC 27853), and *Klebsiella pneumoniae* (ATCC 700603), and a Gram-positive bacterium, *S. aureus* (ATCC 25923). Standard bacterial cultures were obtained from the Department of Microbiology, Faculty of Medicine, University of Ruhuna, Sri Lanka.

Since all lichen extracts showed antimicrobial activity only against *S. aureus* CLSI control strain, 20 MRSA strains (SA 1–20) were used for the second phase of the study along with MSSA as the reference organism. The MRSA strains used in this study were isolated from pus samples obtained for culture and sensitivity testing from patients having skin and soft tissue infections among patients admitted to the Teaching Hospital, Karapitiya, Sri Lanka. Organisms were subcultured for 24 h on blood agar and MacConkey agar, and were confirmed by Gram stain, catalase test, slide and tube coagulase tests, and the zone diameter of cefoxitin (30 *μ*g) on Mueller–Hinton agar at the Department of Microbiology, Faculty of Medicine, University of Ruhuna, Sri Lanka.

#### 2.5.2. Determination of Antibiotic Resistance Profile

Antibacterial activity was determined against CLSI control strain (ATCC 25923) and 20 MRSA strains separately using the disc diffusion assay as previously optimized by us and described by Rathnayake et al. [20]. The crude extract (300 mg/ml), 10-fold, and 100-fold dilutions of each lichen extract, extracted in ethanol, hexane, and water were prepared in 10% DMSO. Bacterial cell suspensions were adjusted to a density of 1 × 10^8^ CFU/ml inoculum using 0.5 McFarland turbidity standards. Each standardized inoculum was distributed evenly on the surface of dried sterile Mueller–Hinton agar plates. Sterile blank filter paper discs (Whattman No. 1, diameter = 6 mm) were impregnated with 10 *µ*lof each lichen extract and were placed on the seeded Mueller–Hinton agar plates. Cefotaxime (30 *μ*g/disk) was used as the positive control against CLSI control strains, vancomycin (30 *μ*g/disc) disk was used as the positive control against MRSA, and 10% DMSO-soaked filter paper disk was used as the negative control in both sets of experiments. The plates were incubated aerobically at 35 ± 2°C for 18–24 h. At the end of the incubation period, the antimicrobial spectrum of lichen extracts was determined for each bacterial species by the measurement of zone size around each disc. Diameters of the zones of inhibition were measured using a Vernier caliper and compared with the controls. Each test was carried out in triplicate, and the average of the diameters was calculated.

#### 2.5.3. Determination of Activity Index (AI) and Relative Percentage Inhibition (RPI)

Activity index (AI) for each lichen extract was calculated using the following formula [21]:

(2) $$\begin{array}{c} \text{AI}=\frac{\text{Inhibition zone diameter of the sample}}{\text{Inhibition zone of the standard}}. \end{array}$$

The relative percentage index was calculated against the positive control using the following formula [21]:

(3) $$\begin{array}{c} \text{RPI}=\frac{100\left(X-Y\right)}{\left(Z-Y\right)}, \end{array}$$

where *X* = total area of inhibition of the lichen extract, *Y* = total area of inhibition of the solvent, and *Z* = total area of inhibition of the standard drug. The total area of the inhibition was calculated using the formula,

(4) $$\begin{array}{c} \text{Area}=\pi r^{2}, \end{array}$$

where *r* = radius of zone of inhibition.

#### 2.5.4. Determination of Minimum Inhibitory Concentration (MIC)

Broth micro-dilution method described in Rathnayake et al. was used to determine the MIC of lichen extracts that gave a positive result for the disk diffusion assay against CLSI control strains and 20 MRSA strains separately [20]. Serial 5-fold dilutions of the lichen extracts were prepared in the 10% DMSO, yielding serial dilutions of the crude lichen extract (300 mg/ml). Bacterial inoculum was prepared in Mueller–Hinton broth, and the turbidity was adjusted to approximately 0.5 McFarland turbidity standards. For the MIC assay, 96-well microtiter plates were used, and 150 *μ*L of plant extract was added to each well of the microplate. Bacterial suspension (50 *μ*L) was added to each well except the negative controls. Vancomycin (MIC ≤ 2 *μ*g/ml) was used as a positive control. DMSO (10%) and plant extracts without bacterial suspension were used as the negative controls. Microtiter plates were incubated at 35 ± 2°C for 24 h. Antimicrobial activity was assessed by the measurement of absorbance at 630 nm using a microplate reader. The lowest concentration (highest dilution) of the extract that prevented visible bacterial growth depending on the absorbance was regarded as the MIC. The assay was done in triplicate for each extract separately, and average absorbance values were used to determine the MIC.

### 2.6. Ethical Approval

Ethical approval for the study was obtained from the Ethical Review Committee, Faculty of Medicine, University of Ruhuna, Sri Lanka.

### 2.7. Statistical Analysis

The mean and the standard error of the mean of all replicates were calculated and data were analysed using one-way ANOVA to compare the mean between groups. *p* < 0.05 was considered as statistically significant.

## 3. Results

In the present study, the chemical composition and antimicrobial activity of three different extracts of two Sri Lankan lichens, *P. rampoddense* and *P. tinctorum* were compared against methicillin-sensitive and methicillin-resistant *S. aureus.*

### 3.1. Identification of Secondary Metabolites of Lichen Extracts

Qualitative analysis of phytoconstituents revealed that both lichens contain saponins, flavonoids, and polyphenols. Also, anthracene glycosides were present in *P. tinctorum* but not in *P. rampoddense*. Proteins, alkaloids, tannins, reducing sugars, cyanogenic glycosides, and cardenolide glycosides were absent in both lichen extracts.

**Figure 2:**
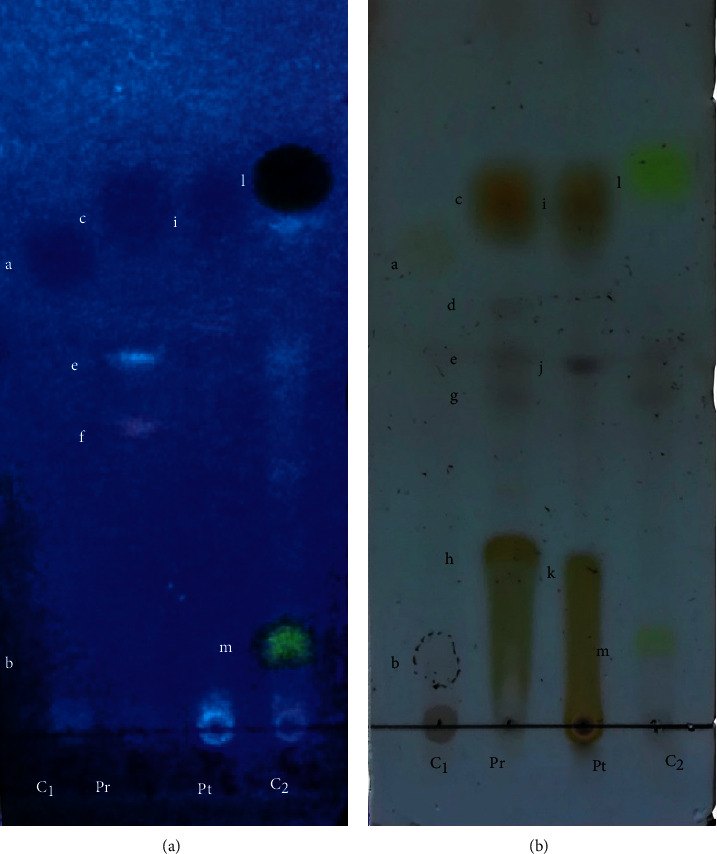
Thin-layer chromatograms developed with the solvent system C (toluene 170: acetic acid 30) visualized under UV light at 366 nm (a) and heated with 10% H_2_SO_4_ (b).

The thin layer chromatograms developed with the medium polar to polar solvent system gave the best separation of compounds in the two lichen extracts indicating that these extracts contain relatively polar phytoconstituents (Figure 2). TLC data indicated the presence of alectorialic acid (h) at Rf. Value of 0.25, atranorin (c) at Rf value of 0.71, and unknown compounds (d, e, f, and g) at Rf values of 0.57, 0.5, 0.42, and 0.45, respectively, in *P. rampoddense* (Pr).

Similarly *P. tinctorum* (Pt) showed the presence of atranorin (i) at Rf value of 0.71, lecanoric acid at Rf value of 0.21(k), and unknown compound (j) at Rf values of 0.49. The control 1 (C_1_) of *Parmelia caperata* showed the presence of usnic acid (a) at Rf value of 0.64, and caperatic acid (b) at Rf value of 0.24. Furthermore, control 2 (C_2_) consisted of chloro-atranorin (At) at Rf values of 0.76 (l) and stictic acid (m) at Rf value of 0.11.

#### 3.1.1. GC-MS Spectrophotometry

**Figure 3:**
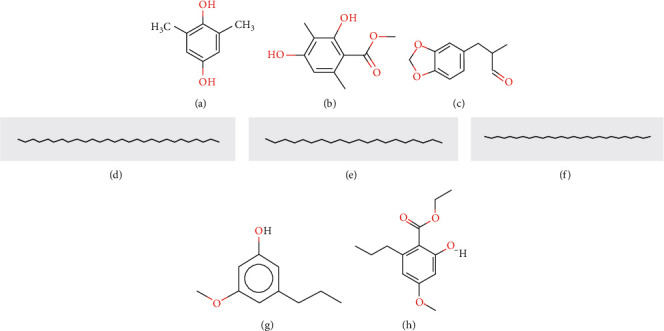
Compounds present in *P. rampoddense*. (a) 1,4-Benzenediol, 2,6-dimethyl. (b) Benzoic acid, 2,4-dihydroxy-3,6-dimethyl-, methyl ester (atraric acid). (c) 1,3-benzodioxole-5-propanal,.alpha.-methyl (helional). (d) Octacosane (C_28_H_58_). (e) Eicosane (C_20_H_42_). (f) Heptacosane (C_27_H_56_). (g) Phenol, 3-methoxy-5-propyl. (h) Ethyl 2-hydroxy-4-methoxy-6-propylbenzoate (Ethyl divaricatinate).

**Figure 4:**
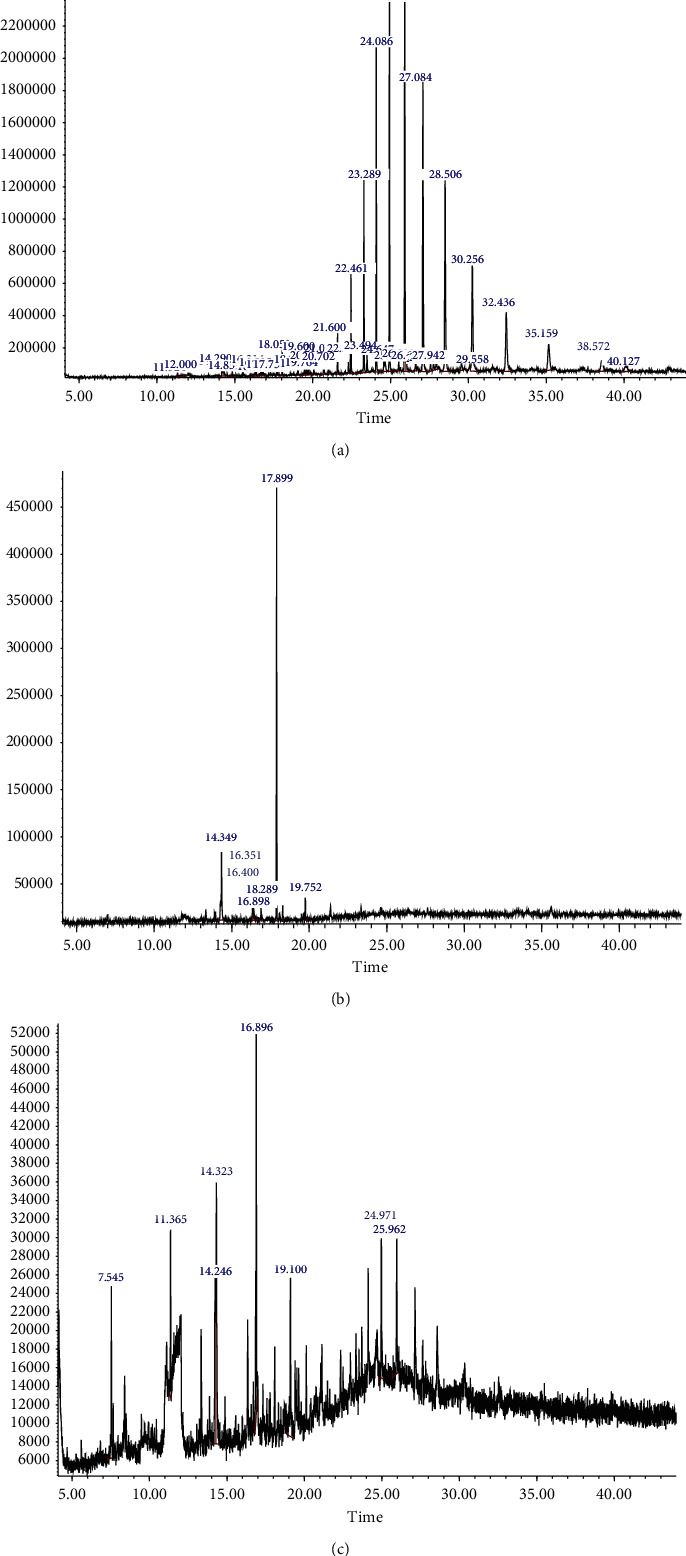
GC-MS chromatograms of crude hexane (a), acetonitrile (b), and chloroform (c) extracts of *P. rampoddense*.

*GC-MS Analysis of P. rampoddense*. GC-MS analysis was carried out on hexane, chloroform, and acetonitrile extracts of *P. rampoddense* whole lichen and 13 major chemical constituents were detected. The active principles with their retention time (RT), Retention Index, molecule name, and percentage of the chemical in the crude extract are presented in Table 1. The molecular structures of major constituents identified from *P. rampoddense* are shown in Figure 3. The chromatograms obtained for hexane, acetonitrile, and chloroform extracts of P. rampoddense showed the presence of 1,4 benzene,2,6-dimethyl, also known as 2,6-dimethylhydroquinone (13.33 min), and benzoic acid, 2,4-dihydroxy-3,6-dimethyl-, methyl ester, also known as atraric acid (16.89 min), in all three solvents. Apart from the above compounds, the GC-MS profiles of hexane extract showed the presence of heptacosane (24.122 min), Nonadecane, 9-methyl (24.97 min), benzeneacetic acid, 2-tetradecyl ester (25.96 min), and octacosane (27.1 min) as the main constituents (Figure 4(a)). Similarly, the GC-MS profile of acetonitrile extract showed the presence of 3-methoy-5-propylphenol (14.34 min) 1,11-dodecadiene (16.40 min), ethyl 2-hydroxy-4-methoxy-6-propylbenzoate (17.89 min), and octadecanoic acid, ethyl ester (19.75 min) (Figure 4(b)). Furthermore, eicosane (14.32 min), tridecane, 3-methyl (7.54 min), octacosane (24.97 min), and tetracosane (25.96 min) were identified in the GC-MS chromatogram of the chloroform extract of *P. rampoddense* (Figure 4(c)). Other constituents were represented by very low percentages.

**Figure 5:**
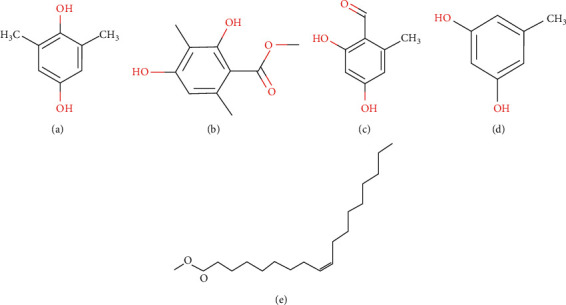
Compounds present in *P. tinctorum*. (a) 1,4-Benzenediol,2,6-dimethyl. (b) Benzoic acid, 2,4-dihydroxy-3,6-dimethyl-, methyl ester (atraric acid). (c) Benzaldehyde,2,4-dihydroxy-6-methyl (O-orsellinaldehyde). (d) 3,5-dihydroxytoluene (orcinol). (e) 9-octadecanoic acid, methyl ester.

**Figure 6:**
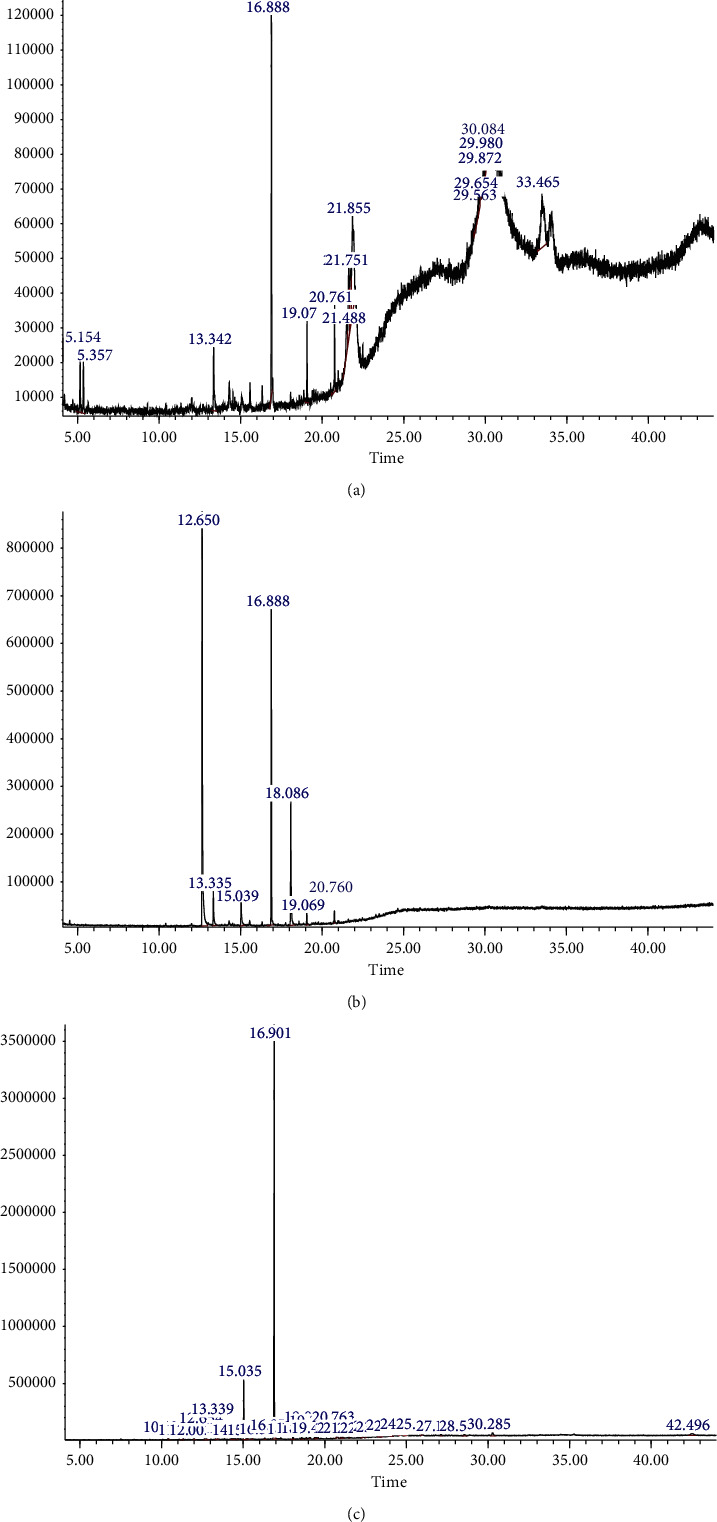
GC-MS chromatograms of crude hexane (a), acetonitrile (b), and chloroform (c) extracts of *P. tinctorum*.

*GC-MS Analysis of P. tinctorum*. GC-MS analysis was carried out on hexane, chloroform, and acetonitrile extracts of *P. tinctorum* whole lichen, and 12 major chemical constituents were detected. The active principles with their retention time (RT), molecule name, and percentage of the chemical in the crude extract are presented in Table 2. The molecular structures of major constituents identified from *P. tinctorum* are shown in Figure 5. The chromatograms of hexane, acetonitrile, and chloroform extracts of *P. tinctorum* showed the presence of 2,6-dimethylhydroquinone (13.33 min), atraric acid (16.9 min), and 9-octadecanoic acid, methyl ester (20.7 min) as major constituents present in all three extracts. Pentadecanoic acid, 14-methyl, methyl ester (19.07 min), and octadecanoic acid (20.98 min) (Figure 6(a)) were identified in the GC-MS chromatogram of the hexane extract of *P. tinctorum*. Similarly, the GC-MS profile of acetonitrile extract also showed the presence of orcinol (12.65 min), o-orsellenaldehyde (15.03 min), 7-hexadecanoic acid, methyl ester (19.06 min) (Figure 6(b)). In addition to the above major compounds, the GC-MS profiles of chloroform extract showed 3,5-dihydroxytoluene (orcinol) (12.65 min), benzaldehyde, 2,4-dihydroxy-6-methyl (o-orsellenaldehyde) (15.03 min) with high percentages (Figure 6(c)). Other constituents were represented by very low percentages.

### 3.2. Determination of Antimicrobial Activity of Lichen Extracts

#### 3.2.1. Antimicrobial Profile of Lichen Extracts Using Agar Disc Diffusion Assay

**Figure 7:**
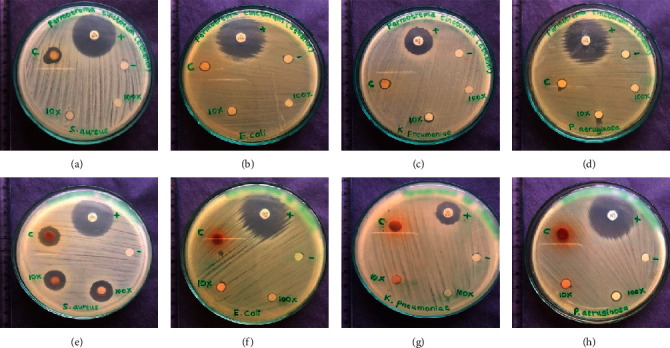
Inhibitory zones obtained for *P. tinctorum* and *P. rampoddense* tested against Gram-positive and Gram-negative bacteria. Top row shows the inhibitory zones of crude ethanol extracts of *P. tinctorum* against (a) *S aureus*, (b) *E coli*, (c) *K pneumonia*, and (d) *P aeruginosa,* and botom row shows the inhibitory zones of crude ethanol extracts of *P.rampoddense* against (e) *S aureus,* (f) *E coli,* (g) *K pneumonia*, and (h) *P aeruginosa*; (+): positive control, cefotaxime (30 *μ*g/disk); (−): negative control, 10% DMSO; (c) crude extract; 10*x*: 10-fold dilution of crude extract; 100*x*: 100-fold dilution of the crude extract.

Ethanol, hexane, and aqueous extracts of *P. tinctorum* and *P. rampoddense* were first subjected to an agar disc diffusion assay to identify the presence of potential antibacterial compounds against both Gram-positive and Gram-negative bacteria. Crude extract and its 10-fold and 100-fold dilutions were prepared, and the inhibitory activity of each extract was quantified by the measurement of the diameter of the zone of inhibition. Measurable inhibitory zones were observed only against the Gram-positive bacterium, MSSA. None of the lichen extracts showed any activity against the Gram-negative bacteria, *Escherichia coli*, *Pseudomonas aeruginosa*, and *Klebsiella pneumoniae. P. tinctorum* gave inhibitory zones only for the ethanol and the hexane extracts but *P. rampoddense* showed inhibitory zones for all three extracts (Table 3, Figure 7). Compared to *P. tinctorum*, which showed an inhibitory zone of 9.4 mm only at 300 mg/ml, *P. rampoddense* showed larger inhibitory zones (14.4, 15.3, 16.5 mm at 300, 30, and 3 mg/ml, respectively) in the presence of all three concentrations of the ethanol extract. However, the inhibitory zones given by the hexane (7.8, 6.5 mm at 300 and 30 mg/ml, respectively) and the aqueous extracts (8.1, 7.9 mm at 300 and 30 mg/ml, respectively) of *P. rampoddense* were comparatively lower (Table 3, Figure 7).

**Figure 8:**
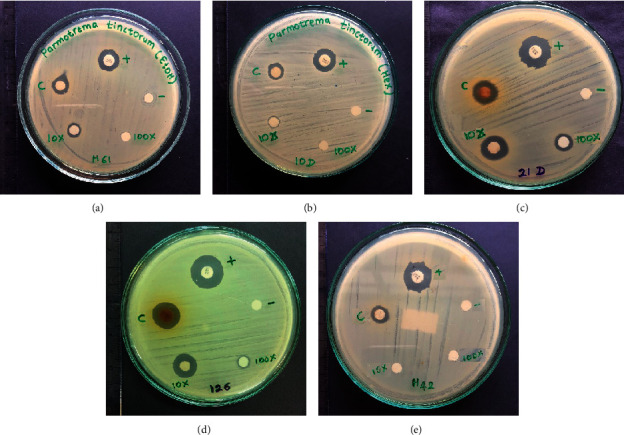
Inhibitory zones of crude extracts of *P. tinctorum:* (a) ethanol extract; (b) hexane extract. Crude extracts of *P. rampoddense*: (c) ethanol extract, (d) hexane extract, and (e) aqueous extract, against different strains of methycillin resistant *Staphylococcus aureus*. The lichen extracts that gave positive results for the disc diffusion assay for some MRSA strains are shown here. (+): positive control, vancomycin (30 *μ*g/disc); (-): negative control, 10% DMSO; (c) crude extract; 10*x*: 10-fold dilution of crude extract; 100*x*: 100-fold dilution of the crude extract.

The same trend was observed against twenty clinical isolates of MRSA. Relatively larger inhibitory zones between 7.9 and 11.4 mm were observed from the undiluted crude ethanol extract of *P. rampoddense*, while ten-fold and hundred-fold dilutions of the ethanol extract gave inhibitory zones between 8.5–12.4 mm and 6.1–8.2 mm, respectively. However, diameters of inhibitory zones of water and hexane extracts were lower ranging from 6.1–7.4 mm and 6.1–8.0 mm, respectively (Table 4, Figure 8). Hexane and aqueous extracts of *P. rampoddense* gave measurable inhibitory zones only at 300 mg/ml. Consistent with the results obtained against MSSA, relatively lower inhibitory zones were observed in the presence of ethanol and hexane extracts of *P. tinctorum*, but the aqueous extract was inactive against MRSA. All results were compared against the standard drug, Vancomycin (30 *μ*g/disc), which showed inhibitory zones of 20.8 mm and 13.53 mm, respectively, against MSSA and MRSA.

#### 3.2.2. Activity Index (AI) and Relative Percentage Inhibition (RPI) of Lichen Extracts

**Figure 9:**
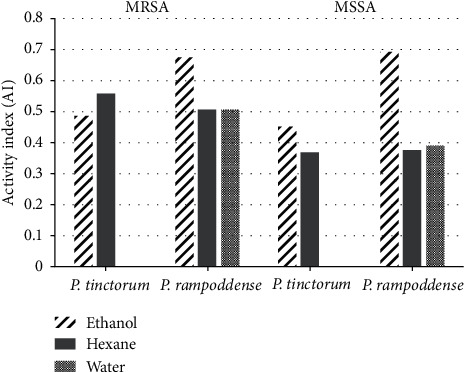
Activity index calculated for the activity of different extracts of *P. tinctorum* and *P. rampoddense* against MRSA and MSSA.

**Figure 10:**
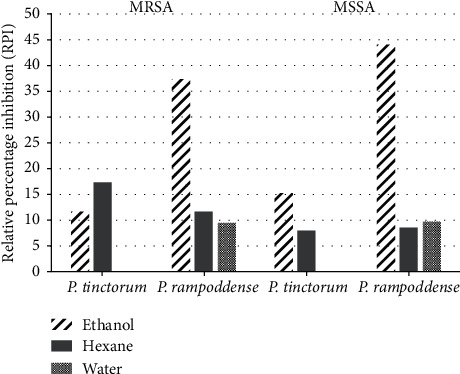
Relative percentage index calculated for the activity of different extracts of *P. tinctorum* and *P. rampoddense* against MRSA and MSSA.

Antibacterial activity of the extracts was compared with the antibacterial activity of the standard drug (positive control), Vancomycin. Ethanol extract of *P. rampoddense* exhibited the maximum AI (Figure 9) as well as the RPI (Figure 10) against MSSA (AI of 0.69, RPI of 44.46%) and MRSA (AI of 0.68, RPI of 37.38) compared to the hexane and aqueous extracts. AI and RPI calculated for *P. tinctorum* were relatively lower in all three extracts compared to *P. rampoddense.*

#### 3.2.3. Minimum Inhibitory Concentration of Lichen Extracts

As expected, results of the broth microdilution assay confirmed that MSSA (ATCC 25923) was more susceptible against ethanol extract of *P. rampoddense* with the lowest MIC of 0.0192 mg/ml than the MRSA, which showed a MIC of 0.096 mg/ml for 25% of clinical isolates (Table 5). MIC ranges obtained for the ethanol and hexane extracts of *P. tinctorum* were 2.4–12 mg/ml, whereas MIC ranges obtained for the hexane, ethanol, and aqueous extracts of *P. rampoddense* were 12–60, 0.096–2.4, and 2.4–60 mg/ml, respectively. Out of the 20 MRSA isolates used in the study, 60% and 90% were inhibited by the presence of 2.4 mg/ml of ethanol and hexane extracts of *P. tinctorum*, respectively. However, hexane extract of *P. rampoddense* was less effective in the inhibition of MRSA isolates in which 75% of the isolates were inhibited at 60 mg/ml and 25% of the isolates were inhibited at 12 mg/ml. Comparatively, the aqueous extract of *P. rampoddense* showed better activity in which 25%, 55%, and 20% of the MRSA isolates were inhibited by 60, 12, and 2.4 mg/ml of the lichen extract, respectively. The best activity was shown by the ethanol extract of *P. rampoddense* where 25% of the MRSA isolates were inhibited by 0.096 mg/ml of the extract, whereas 25% and 50% of the isolates were inhibited by 0.48 mg/ml and 2.4 mg/ml of the same extract, respectively.

## 4. Discussion

Resistant microbial populations emerged in a higher magnitude in the recent past due to inappropriate and irrational use of antibiotics. Among the mechanisms suggested, alteration of target sites, enzymatic degradation, and active efflux of drugs are some of the strategies used by the pathogenic bacteria to develop inherent resistance developed against antibiotics. Unfortunately, Food and Drug Administration (FDA) has approved only two antibacterial agents for use in humans from 2008 to 2016, compared to the 16 drugs approved from 1983 to 1987 [22]. Therefore, novel bioactive preparations from lichens may offer a plethora of interesting possibilities to combat drug resistance.

Relatively, a limited number of studies have thus far explored the biological activities of lichens worldwide [23]. Antibacterial activities of lichens were first reported in 1944 by Burkholder et al. [24] and since then, several studies have emerged on their protective effect against both Gram-negative and Gram-positive bacteria [24]. Results from previous studies have suggested that it is much difficult for bacteria to develop resistance against multiple and chemically complex phytochemicals present in plant extracts including lichens [25]. It is believed that secondary metabolites of plants were optimized during evolution so that they can interfere with molecular targets in herbivores and microbes and act as defense mechanisms [26].

This study provides data based on the first broad-scale screening of antimicrobial activity of two lichens collected from Belihuloya, Sri Lanka, against Gram-negative and Gram-positive bacteria including twenty clinically isolated MRSA. In the preliminary study we conducted, it was reported that lichens extracted to three different solvents of varying polarities were not active against any of the Gram-negative bacteria, *Escherichia coli, Pseudomonas aeruginosa*, and *Klebsiella pneumoniae*. Our results are comparable to the results reported previously by other authors [27–30]. This can be ascribed to the sensitivity and differences in the permeability of the cell wall of bacteria. The cell wall of Gram-positive bacteria is made up of peptidoglycans and teichoic acids, whereas the cell wall of Gram-negative bacteria are made up of peptidoglycans, lipopolysaccharides, and lipoproteins, which offers more resistance than the Gram-positive bacteria [31].

We determined the antibacterial activity of *P. tinctorum* and *P. rampoddense* against the standard strain of *S. aureus* (ATCC 25923), and 20 clinically isolated strains of MRSA (MRSA 1–20). These bacteria were used as test microorganisms due to their clinical significance, which imposes serious threats against individuals. According to the results obtained, MSSA was more sensitive than MRSA against both *P. tinctorum* and *P. rampoddense*. Our results against *S. aureus* are comparable to several other studies reported previously by multiple authors [32–34]. We also used crude aqueous, ethanol, and hexane extracts of all lichens for the study. Crude extracts are often used in traditional systems of medicine, such as Ayurveda, commonly practiced in Sri Lanka, due to their belief in the synergistic effect of a multitude of phytoconstituents, which may give rise to the observed bioactivity. It is also believed that the separation of these compounds may alter the activity of individual compounds, which may be below the sensitivity threshold [35].

According to the results, we found that all hexane and ethanol extracts of the tested lichen species, except the aqueous extracts, were active against both methicillin-sensitive and methicillin-resistant *S. aureus* at 300 mg/ml but some were even active at lower concentrations such as 30 and 3 mg/ml (Tables 3 and 5). Although both hexane and ethanol extracts demonstrated activity against all the MRSA strains, the ethanol extract was more active than the hexane extract (Table 4). Ethanol extract of *P. rampoddense* showed the highest activity index (Figure 9) and relative percentage index (Figure 10) and also the lowest MIC values of 0.096 mg/ml (Table 4) against five isolates of methicillin-resistant *S. aureus*. Hence, it can be concluded that the polarity of the solvent used during the extraction process determines which phytoconstituents were extracted that has a direct effect on the antimicrobial potential of the lichens under study.

Different types of secondary metabolites, such as depsides, depsidones, hydroxybenzoic acid derivatives, anthraquinones, dibenzofurans, and aliphatic acids, are produced by lichens and are deposited in the upper cortex or fruiting bodies on the thallus in liquid or crystal form [31]. The antimicrobial activity could be correlated with the secondary metabolites of *P. rampoddense*, which is composed of alectorialic acid and atranorin as the main substances as supported by our TLC results (Figure 2). A significant amount of Atraric acid detected by GC-MS is derived from the well-known lichen compound atranorin [36]. Furthermore, the hexane extract of the lichen indicated significant quantities of n-alkanes and methyl esters of fatty acids similar to *Xanthoria parietina* species [37]. It is a known fact that relatively unstable lichen substances are degraded in the lichen itself or *in vitro* in their extracts at room temperature and Zakeri et al. [38] have shown that alectorialic acid is a relatively unstable substance which can be degraded to other compounds at room temperature. Therefore, degradation products have been reported in the GC-MS analysis. Lichen acids, atranorin, and lecanoric acid were detected in the TLC profile of *P. tinctorum* and the GC-MS analysis confirmed the presence of atraric acid, orcinol, and O-orsellinaldehyde among the major constituents. Previous studies have also detected O-orsellinaldehyde and orcinol as bioactive derivatives obtained from lecanoric acid [38–40]. Similar antimicrobial activities were reported by Gomes et al. for the lecanoric acid isolated from the lichen, *P. tinctorum* [41]. Alectorialic acid and atranorin were among the several metabolites with antimicrobial activities identified by Pilzk et al. in the lichens, *Ramalina* and *Usnea* [31]. According to Jayaprakasha and Rao, phenolic constituents, including methyl orsenillate, orsenillic acid, atranorin and lecanoric acid, were extracted from the lichen *Parmotrema stuppeum* [31]. Atranorin, usnic acid, norstictic acid, protoacetraric acid, atranol, lecanoric acid, stictic acid, and divericatic acids extracted from lichens, *Melanelia subaurifera* and *Melanelia fuliginosa*, were also reported to possess antibacterial activities [42]. In another study, Rankovic et al. also reported that atranorin exhibited antimicrobial activities against *S. aureus, B. subtilis*, and *E coli* [43]

Rajan et al. who determined the antimicrobial activity of acetone extracts of *P. rampoddense* and *P. tinctorum* against *S. aureus* (ATCC 29213) also detected the presence of alectorialic acid and atranorin in both species [44]. Although they investigated the antimicrobial effect of both acetone and methanol extracts, none of the methanol extracts were effective against *S. aureus* (ATCC 29213). Interestingly, in our study, ethanol extracts of both lichens, which also have a higher polarity comparable to methanol, showed the highest antibacterial activity even at 3 mg/ml (*P. rampoddense).* Rajan et al. also revealed that the MIC of acetone extracts of both lichens were 0.5 mg/ml against *S. aureus* (ATCC 29213) [44]. We revealed that *P. rampoddense* ethanol extract at 0.0192 mg/ml was effective against the CLSI control strain of *S aureus* (ATCC 25923). The same lichen extract was also effective against all MRSA strains with MIC between 0.096 and 2.4 mg/ml. Variation in the results reported for *P. rampoddense* and *P. tinctorum* between the two research groups may be due to a combination of factors, including the extraction of different lichen species, type of extract and its concentration, the solvent used for extraction, and the specific bacterial strain [45]. The differences in results between the two studies could also be due to certain adaptations and modifications that could take place for the survival of the species in different climatic conditions and geographical locations. Specific factors that influence the antimicrobial properties of lichen extracts warrant further investigations.

Since we have identified several chemical constituents already proven to be having antimicrobial properties in the two lichen species investigated, it can be assumed that the lichen crude extracts were active against the test organisms due to the probable mechanisms of those secondary metabolites. It is also proven that multiple components in a crude extract act at different sites, thereby contributing to the overall activity of the extract. The lichen extracts may exert their anti-microbial activity by altering the key events in the pathogenesis and not just by killing the bacteria itself [46]. Such key processes may include the inhibition of cell wall synthesis, inhibition of protein synthesis, alteration of the cell membrane integrity, inhibition of nucleic acid synthesis, and anti-metabolite activity [47]. The antibiotic activity could also be correlated with the mechanism of action of various lichen acids, which could inhibit oxidative phosphorylation. As such events happen, oxygen consumption, electron transport chain, and other mitochondrial functions may also be inhibited, leading to cell death [48]. Therefore, it is imperative to investigate the mechanism of action of active constituents of *P. rampoddense* and *P. tinctorum* and optimize their activity to develop them as potential antimicrobial agents in the future.

## 5. Conclusion

Our study provides evidence that the two lichens, *P. rampoddense* and *P. tinctorum*, collected from Belihuloya, Sri Lanka, represent potentially important source of future antimicrobial drugs. Our research provides evidence that all extracts showed a significant inhibitory activity but ethanol extract of *P. rampoddense* is more effective than *P. tinctorum* at MIC 0.0192 mg/ml against methicillin-sensitive *Staphylococcus aureus* and at the minimum MIC of 0.096 mg/ml against methicillin-resistant *Staphylococcus aureus*. Among the chemical constituents identified, alectorialic acid, lecanoric acid, atranorin, atraric acid, orcinol, and O-orsellinaldehyde may be responsible for the antimicrobial activity of the two lichens used in the study. These two lichen species merit further investigation to determine their mechanism of action against bacterial pathogens and also their levels of cytotoxicity against normal cells.

## Figures and Tables

**Table 1:** Chemical constituents identified in abundance in the GC-MS analysis of *P. rampoddense*.

	Name of the compound	Hexane			Acetonitrile			Chloroform		
		RT (min)	RI	% area	RT (min)	RI	% area	RT (min)	RI	% area
1	Limonene	—	—	—	7.01	1035	1.145	—	—	—
2	Benzene, 1,3-diethyl	7.421	1055	0.271	—	—	—	—	—	—
3	Tridecane,3-methyl	7.543	1061	0.902	—	—	—	7.544	1061	4.431
4	Dodecane,1-ido	11.365	1284	0.523	—	—	—	—	—	—
5	1,4-Benzenediol,2,6-dimethyl	13.337	1425	0.299	13.338	1425	1.794	13.337	1425	3.234
6	Eicosane	14.326	1501	0.999	—		—	14.323	1501	9.651
7	3-Methoxy-5-propylphenol	—	—	—	14.349	1503	0.12	—	—	—
8	1,11-Dodecadiene	—	—	—	16.401	1676	2.147	—	—	—
9	Heptacosane	16.856	1717	0.307	—		—	16.856	1717	2.654
10	Atraric acid	16.899	1721	0.308	16.898	1721	2.193	16.897	1721	7.622
11	Ethyl 2-hydroxy-4-methoxy-6-propylbenzoate	—	—	—	17.899	1908	50.357	—	—	—
12	Palmitic acid	19.404	1982	0.676	—		—	—	—	—
13	Octadecanoic acid, ethyl ester	—	—	—	19.752	1997	2.594	—	—	—
14	Octacosane	21.638		0.323				24.971		4.160
15	Tetradecane	22.499		1.111				—		—
16	Tetracosane	23.325		2.751				25.962		4.432
17	Heptacosane	24.122		6.123				24.121		3.69
18	Nonadecane,9-methyl	24.974		9.799						
19	Benzeneacetic acid, 2-tetradecyl ester	25.964		12.110						
20	Eicosane, 7-hexyl	26.680		0.462						
21	Octacosane	27.145		13.877						
22	Squalene	27.637		1.804						
23	Heptacosane	28.580		10.263						
24	Benzeneacetic acid, 2-tetradecyl ester	30.351		7.912						
25	Octacosane	35.305		3.602						

RT-Retention time (min.), RI–Nonisothermal retention index calculated using Van Den Dool and Kratz method using C_8_–C_20_ alkane series. % Area is expressed as percentage of the peak area to the total peak area.

**Table 2:** Chemical constituents identified in abundance in the GC-MS analysis of *P.tinctorum*.

	Name of the compound	Hexane			Acetonitrile			Chloroform		
		RT (min)	RI	% area	RT (min)	RI	% area	RT (min)	RI	% area
1	Benzaldehyde,2,4,-dimethyl	—	—	—	10.409	1223	0.265	10.409	1223	0.73
2	Hexadecane,2,6,10,14-tetramethyl	—	—	—	—	—	—	11.337	1283	0.228
3	3,5-Dihydroxytoluene	—	—	—	12.65	1374	30.086	12.654	1375	2.572
4	1,4-Benzenediol, 2,6-dimethyl	13.342	1425	1.915	13.335	1425	2.939	13.339	1425	4.084
5	Chloroatranol (3-chloro-2,6-dihydroxy-4-methyl benzaldehyde)	—	—	—	—	—	—	14.44	1511	0.633
6	Benzaldehyde,2,4-dihydroxy-6-methyl	—	—	—	15.039	1560	2.758	15.035	1560	11.671
7	Atraric acid	16.888	1720	6.723	16.888	1720	16.422	16.901	1721	52.174
8	7-Hexadecanoic acid, methyl ester	—		—	19.069	1966	0.685	18.879	1956	0.478
9	Pentadecanoic acid,14-methyl,methyl ester	19.07	1966	1.436	—	—	—	19.073	1966	1.616
10	*n*-Hexadecanoic acid	—	—	—	—	—	—	19.392	1982	1.402
11	9-Octadecanoic acid, methyl ester	20.761	—	1.932	20.76	-	0.696	20.763	—	2.058
12	Octadecanoic acid	20.92	—	0.702	—	—	—	20.983	—	0.285

RT, retention time (min.), RI, –nonisothermal retention index calculated using Van Den Dool and Kratz method using C_8_–C_20_ alkane series. %, area is expressed as percentage of the peak area to the total peak area.

**Table 3:** Comparison of zone diameters of the disc diffusion assay performed on *P.tinctorum* and *P. rampoddense* against MSSA (ATCC 25923) and 20 clinical isolates of MRSA.

Diameters of the inhibitory zones (mm)					
Extract	Concentration (mg/ml)	MSSA (ATCC 25923)^∗^		MRSA^#^#	
		*P. tinctorum*	*P.rampoddense*	*P. tinctorum*	*P. rampoddense*
Ethanol	300	9.4 ± 0.1	14.4 ± 0.8	7.13 (6.2–9.6), *n* = 17	9.61 (7.9–11.4), *n* = 20
	30	—	15.3 ± 0.6	7.11 (6.1–11.1), *n* = 16	10.38 (8.5–12.4), *n* = 20
	3	—	16.5 ± 0.4	—	7.03 (6.1–8.2), *n* = 15

Hexane	300	7.7 ± 0.2	7.8 ± 0.1	7.24 (6.3–9.1), *n* = 18	6.76 (6.1–7.4), *n* = 18
	30	7.5 ± 0.1	6.5 ± 0.1	6.78 (6.4–7.1), *n* = 5	—
	3	6.8 ± 0.3	—	—	—

Aqueous	300	—	8.1 ± 0.2		7.28 (6.1–8.0), *n* = 13
	30	—	7.9 ± 0.1	—	—
	3	—	—	—	—

Positive control	30 *μ*g/disk	20.8 ± 0.4	13.53 ± 0.23		

Negative control	10%	—	—		

^∗^Data are expressed as mean ± SEM. ^#^Data are expressed as mean (Min-Max) of the 20 clinical isolates of MRSA. *n* denotes the number of MRSA isolates which gave a measurable diameter. Vancomycin was used as the positive control. 10% DMSO was used as the negative control. – denotes a measurable diameter was not observed.

**Table 4:** Zone diameters of the disc diffusion assay performed on *P. tinctorum* and *P. rampoddense* against 20 clinical isolates of MRSA.

Organism	*P. tinctorum*				*P. rampoddense*					
	Ethanol		Hexane		Ethanol		Hexane		Aqueous	
	(+) control	Crude	(+) control	Crude	(+) control	Crude	(+) control	Crude	(+) control	Crude
1	11.9	5.2	10.9	6.9	14.6	9.9	13.7	7	13.9	7.4
2	14	9.6	14.3	7.3	14.9	11.4	13.5	6.7	13.9	0
3	16.5	6.2	15.9	7.2	14.9	10.6	12.9	6.5	12.4	5.3
4	17.3	7.9	14.7	7.1	13.3	8.9	13	7.2	13	8
5	12.8	10	11.1	7	13.9	9.1	13	7.3	13.2	6
6	12.4	6.6	11	5.7	12.9	10.2	13.2	5.5	12.7	7.3
7	14.8	5.4	10.8	6.3	14.2	9	12.5	6	14.3	8
8	13.5	6.8	11.4	7	14.4	8.2	13.3	6.1	13.4	7.3
9	15	6.1	14	6.9	16.6	10	13.5	6.2	14.4	7
10	13.4	7.1	11.2	7.1	15.7	8.5	13.1	7.3	12.5	6
11	14.9	7.4	14.5	6.4	14.4	9.4	13	7.2	14.6	7.2
12	13.4	6.2	15	8.7	16	10.6	13.7	7.4	14.3	7
13	16.5	8.1	11.4	7.5	11.8	9.8	13.9	6.8	12.9	5.6
14	16	6.3	12.6	9.1	14.7	10	13	6.9	13.4	6.7
15	10.8	6.2	9.6	7	13.8	7.9	12.5	5.8	13.8	5.5
16	12.1	7.4	11.7	7.2	12.6	9.8	12.4	6	13.3	7
17	15.2	5.2	16.8	7	15.6	11.1	13.1	6.4	12.9	5.8
18	13	6.5	14	7.4	13.8	9.4	13	6.5	13.6	6.1
19	13.1	6.6	11.7	6	12.8	8.9	13.2	7	13.4	7.8
20	16.4	6.2	11.8	7.3	13	9.4	13.3	7.2	13.4	7.8
Average	14.15	6.85	12.72	7.10	14.19	9.60	13.14	6.65	13.46	6.44

Vancomycin was used as the positive control. 10% DMSO was used as the negative control and they did not show any measurable zones. All crude extracts were at 300 mg/ml. Some ethanol and hexane extracts of *P. tinctorum* and *P. rampoddense* gave measurable zones at 30 and 3 mg/ml concentrations but they are not shown here.

**Table 5:** Minimum inhibitory concentration (MIC) of different lichen extracts determined by broth microdilution assay against MSSA (ATCC 25923) and 20 clinical isolates of MRSA.

Bacterial strain	Minimum inhibitory concentration (MIC) (mg/ml)				
	Hexane extract		Ethanol extract		Aqueous extract
	*P. tinctorum*	*P. rampoddense*	*P. tinctorum*	*P. rampoddense*	*P. rampoddense*
MSSA	2.4	12	2.4	0.0192	2.4
MRSA 1	12	12	2.4	2.4	60
MRSA 2	12	60	12	0.096	60
MRSA 3	2.4	60	2.4	0.096	12
MRSA 4	2.4	60	2.4	0.48	60
MRSA 5	2.4	12	12	2.4	12
MRSA 6	2.4	60	12	2.4	12
MRSA 7	2.4	60	12	0.096	12
MRSA 8	2.4	60	2.4	2.4	12
MRSA 9	2.4	60	2.4	2.4	12
MRSA 10	2.4	12	12	0.48	12
MRSA 11	2.4	60	2.4	2.4	12
MRSA 12	2.4	60	2.4	0.48	2.4
MRSA 13	2.4	12	2.4	2.4	2.4
MRSA 14	2.4	60	2.4	0.096	12
MRSA 15	2.4	60	2.4	2.4	12
MRSA 16	2.4	60	12	0.096	2.4
MRSA 17	2.4	12	12	2.4	60
MRSA 18	2.4	60	2.4	0.48	60
MRSA 19	2.4	60	12	2.4	2.4
MRSA 20	2.4	60	2.4	0.48	12

## Data Availability

The datasets used and/or analysed during the current study are available from the corresponding author on reasonable request.

## Abbreviations

MRSA: Methicillin-resistant *Staphylococcus aureus*
MSSA: Methicillin-sensitive *Staphylococcus aureus*
CLSI: Clinical & laboratory standards institute
*P. rampoddense*: *Parmotrema rampoddense*
*P. tinctorum*: *Parmotrema tinctorum*
TLC: Thin later chromatography
GC-MS: Gas chromatography mass spectrophotometry
ATCC: American type culture collection
DMSO: Dimethyl sulfoxide
AI: Activity index
RPI: Relative percentage inhibition
MIC: Minimum inhibitory concentration
Rf: Retention factor.
